# Clinicopathological characteristics and prognosis of uterine sarcoma: a 10-year retrospective single-center study in China

**DOI:** 10.1186/s13000-024-01517-x

**Published:** 2024-07-05

**Authors:** Jin-feng Wang, Chen Li, Jing-yi Yang, Yue-ling Wang, Jing Ji

**Affiliations:** 1 Department of Obstetrics and Gynecology, the First Affiliated Hospital of Xi’an Jiaotong University, 277 Yanta West Road, Xi’an, 710061 Shaanxi China; 2 Medical Records Room, the First Affiliated Hospital of Xi’an Jiaotong University, 277 Yanta West Road, Xi’an, 710061 Shaanxi China

**Keywords:** Uterine sarcoma, Clinicopathological characteristics, Prognosis, Survival, Chinese patients

## Abstract

**Background:**

Uterine sarcoma is a rare and heterogeneous gynecological malignancy characterized by aggressive progression and poor prognosis. The current study aimed to investigate the relationship between clinicopathological characteristics and the prognosis of uterine sarcoma in Chinese patients.

**Methods:**

In this single-center retrospective study, we reviewed the medical records of 75 patients with histologically verified uterine sarcoma treated at the First Affiliated Hospital of Xi’an Jiaotong University between 2011 and 2020. Information on clinical characteristics, treatments, pathology and survival was collected. Progression-free survival (PFS) and overall survival (OS) were visualized in Kaplan-Meier curves. Prognostic factors were identified using the log-rank test for univariate analysis and Cox-proportional hazards regression models for multivariate analysis.

**Results:**

The histopathological types included 36 endometrial stromal sarcomas (ESS,48%), 33 leiomyosarcomas (LMS,44%) and 6 adenosarcomas (8%). The mean age at diagnosis was 50.2 ± 10.7 years. Stage I and low-grade accounted for the majority. There were 26 recurrences and 25 deaths at the last follow-up. The mean PFS and OS were 89.41 (95% CI: 76.07-102.75) and 94.03 (95% CI: 81.67-106.38) months, respectively. Univariate analysis showed that > 50 years, post-menopause, advanced stage, ≥ 1/2 myometrial invasion, lymphovascular space invasion and high grade were associated with shorter survival (*P* < 0.05). Color Doppler flow imaging positive signals were associated with shorter PFS in the LMS group (*P* = 0.046). The ESS group had longer PFS than that of the LMS group (99.56 vs. 76.05 months, *P* = 0.043). The multivariate analysis showed that post-menopause and advanced stage were independent risk factors of both PFS and OS in the total cohort and LMS group. In the ESS group, diagnosis age > 50 years and high-grade were independent risk factors of PFS, while high-grade and lymphovascular space invasion were independent risk factors of OS.

**Conclusion:**

In Chinese patients with uterine sarcoma, post-menopause and advanced stage were associated with a significantly poorer prognosis. The prognosis of ESS was better than that of LMS. Color Doppler flow imaging positive signals of the tumor helped to identify LMS, which needs to be further tested in a larger sample in the future.

## Introduction

Uterine sarcoma is a rare and aggressive heterogeneous malignant tumor originating from the mesodermal tissues (muscle and supportive tissues) [1]. It is characterized by nonspecific clinical presentations, high recurrence rates and poor prognosis, accounting for about 1% of female genital tract malignancies and 3–7% of uterine cancers [2]. The incidence of uterine sarcoma increases with age and is reported to be about 6.4 per 100,000 in women aged above 50 years in America [3].

According to the traditional histological classification, uterine sarcoma mainly included carcinosarcoma (CS), leiomyosarcoma (LMS), endometrial stromal sarcoma (ESS), undifferentiated sarcoma (UUS) and other less frequent histological subtypes, such as adenosarcoma. In 2009, the International Federation of Gynecology and Obstetrics (FIGO) revised the staging system and reclassified CS as endometrial cancer due to its similar dedifferentiated or metaplastic form to endometrial cancer [2]. The new uterine sarcoma classification mainly contains three pathological subtypes: LMS, ESS, adenosarcoma and undifferentiated endometrial sarcoma, of which LMS is the most common [4].

Diagnosis of uterine sarcoma is generally difficult before surgery because of nonspecific symptoms, such as irregular vaginal bleeding, abdominal or pelvic mass and pain, or even no symptom [4, 5]. Ultrasonography, magnetic resonance imaging, computed tomography and cancer antigen 125 (CA125) level are useful preoperative diagnostic methods. However, distinguishing uterine sarcoma from benign uterine lesions such as fibroids is difficult due to the lacking specific symptoms or diagnostic techniques, resulting in high misdiagnosis rates, which may lead to serious consequences [6, 7].

There is no standardized treatment for uterine sarcoma due to its rarity and heterogeneity. Early-stage uterine sarcoma is mainly treated by surgery according to different pathological types, including total hysterectomy with bilateral salpingo-oophorectomy (TH-BSO) [8]. For advanced-stage uterine sarcoma, complete cytoreduction is embraced as the most effective therapy [8]. The effects of lymphadenectomy and adjuvant treatments remain inconclusive, contributing to the dilemma in managing the disease. The reported 5-year survival rate was 45–50% for stage I-II and decreased to 0–15% for advanced stages [9]. Importantly, there are suggestions that the efficiency of treatments can be different among racial populations, indicating the need for therapeutic-tailored strategies [10].

The present study aimed to evaluate the relationship between the prognostic factors, such as clinicopathological characteristics, surgical practices, adjuvant therapies and survival with uterine sarcoma at our institution. The objectives of our evaluation were to increase understanding and individualize the treatment of the disease, to review the data for potential guidelines for therapeutic decisions, and to compare survival outcomes with the different prognostic factors in this rare group of heterogeneous malignancies.

## Materials and methods

### Case inclusion

This study utilized a retrospective design to evaluate patients with histologically verified uterine sarcoma, who were diagnosed and treated at the Department of Gynecology and Obstetrics of the First Affiliated Hospital of Xi’an Jiaotong University from January 2011 to December 2020. The inclusion criteria included: (1) pathologically confirmed uterine sarcoma; (2) complete clinical, pathological and follow-up information. The exclusion criteria included: (1) other cancers excluding breast cancer; (2) metastatic or other sites’ sarcomas; (3) endometrial carcinosarcoma; (4) currently pregnant; (5) histories of preoperative chemotherapy or radiotherapy; (6) lost to follow-up. Finally, a total of 75 cases were included in this study. The requirement for written informed consent from participants was waived due to the retrospective nature of the study. Verbal informed consent was obtained from surviving patients and the family members of deceased patients during phone call follow-up. They were approved by the ethics committee of the First Affiliated Hospital of Xi’an Jiaotong University (No. XJTU1AF2023LSK-275).

### Data extraction

The following clinical data were extracted from medical records: age at diagnosis, menopausal status, FIGO stage, tumor size, color Doppler flow imaging (CDFI), surgery type and time, lymph node metastasis, adjuvant therapy, histological type, histological grade (low grade indicates high differentiation, while high grade indicates low differentiation), myometrial invasion (< 1/2, ≥ 1/2), and lymphovascular space invasion (LVSI). The surgical staging was defined by the FIGO 2009 staging system.

### Outcome measures

Survival information was acquired by telephone and medical records. Progression-free survival (PFS) was defined from the first time for surgery to that of disease progression or recurrence. Overall survival (OS) was defined as the time from initial surgery to death from all causes [11]. The analysis cut-off date was set at July 5, 2022, with survival times calculated in months.

### Statistical analysis

Categorical variables were presented as frequencies (percentages), and continuous variables as means ± standard deviations (SD), or medians and ranges. Prognostic factors for PFS and OS were examined by the log-rank tests for univariate analysis. Significant factors in univariate analysis and clinically significant indicators were included in the Cox regression model for further multivariate analysis.

Survival curves were plotted using the Kaplan-Meier method. Differences among curves were analyzed by the log-rank tests. *P* < 0.05 was considered as statistically significant. All statistical analyses were performed using SPSS version 22.0 (IBM Corp., Armonk, NY, USA).

### Results general information of patients

**Fig. 1:**
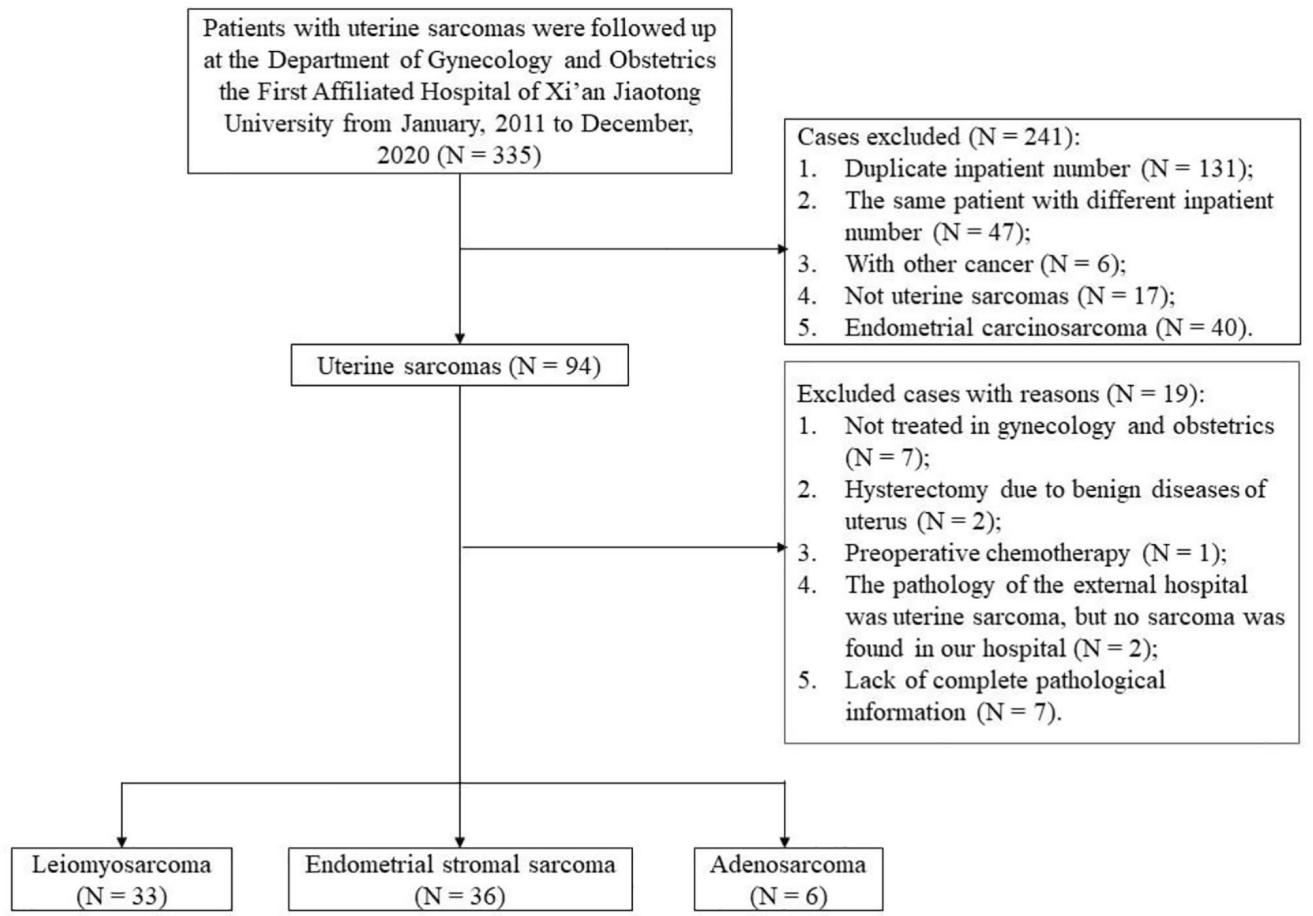
Flow chart of the patient selection process

Over the 10-year study period, a total of 335 uterine sarcoma cases were confirmed and retrieved from the Department of Gynecology and Obstetrics of the First Affiliated Hospital of Xi’an Jiaotong University. Following stringent adherence to our inclusion and exclusion criteria, 75 cases were enrolled ultimately, including ESS (*n* = 36), LMS (*n* = 33) and adenosarcoma (*n* = 6). Detailed process of case inclusion was shown in Fig. 1. The median follow-up period was 50 months (range: 4.7-132.3 months).

Table 1 showed the general information of patients. The mean age at diagnosis was 50.2 ± 10.7 years old (range: 31–81 years), which served as the threshold to categorize patients into two groups. Forty-nine (65.3%) patients were premenopausal and 26 (34.7%) were post-menopausal. A tumor size of over 5 cm was a prerequisite for surgery and was used as a critical point in this study. Among 62 cases with complete data on CDFI, 39 (62.90%) had positive signals. Surgical treatment was administered to 73 patients, with 68 (93.2%) patients, having total hysterectomy with TH-BSO, and 5 (6.8%) patients having total hysterectomy alone. Lymphadenectomy was performed in 24 patients, and only one LMS patient had lymph node metastasis with 9 months’ survival. Based on FIGO staging, most patients (81.33%) were diagnosed as stage I. Most patients had adjuvant chemotherapy with 4–6 cycles after surgery (76.71%, 56/73) according to postoperative pathology including FIGO stage, histological grade, myometrial invasion, lymph node metastasis, LVSI and so on. The most common chemotherapy combination was paclitaxel and platinum (PT) (64.29%, 36/56), followed by ifosfamide + epirubicin + cisplatin (IAP) (26.79%, 15/56). A patient diagnosed with ESS received progesterone therapy without chemotherapy after surgery, with an OS of 47.5 months. For the remaining two patients, one patient refused any treatment because of old age and advanced stage, with an OS of 4.7 months. Another patient received radio-chemotherapy, with an OS of 15 months.

**Table 1:** General information about patients

	total (*n* = 75)	ESS (*n* = 36)	LMS (*n* = 33)	Adenosarcoma (*n* = 6)
**Age at diagnosis (years)**				
≤ 50	42	21	18	3
>50	33	15	15	3
Mean ± standard deviation	50.2 ± 10.7	49.9 ± 11.9	51.2 ± 9.8	46.8 ± 8.9
Median (range)	48 (31–81)	48 (31–81)	48 (35–73)	47 (34–57)
**Menopausal state**				
premenopausal	49	23	23	3
postmenopausal	26	13	10	3
**CDFI**				
no	23	11	11	1
yes	39	17	17	5
miss	13	8	5	0
**Tumor size (cm)**				
<5	14	9	1	4
≥ 5	48	21	25	2
miss	13	6	7	0
**FIGO Stage**				
I	61	31	24	6
II-IV	11	5	6	0
miss	3	0	3	0
**Histological grade**				
low	35	21	13	1
high	23	12	8	3
miss	17	3	12	2
**Myometrial invasion**				
<1/2	28	15	9	4
≥ 1/2	33	14	17	2
miss	14	7	7	0
**LVSI**				
no	66	32	28	6
yes	6	3	3	0
miss	3	1	2	0
**Treatment plan**				
no treatment	1	0	1	0
surgery	17	9	6	2
surgery + adjuvant therapy	56	27	25	4
adjuvant therapy	1	0	1	0
**Lymphadenectomy**				
no	49	25	21	3
yes	24	11	10	3
**Lymph node metastasis**				
no	23	11	9	3
yes	1	0	1	0
**Chemotherapy regimen**				
Paclitaxel + platinum	36	16	17	3
Ifosfamide + epirubicin + Cisplatin	15	7	7	1
other	5	3	2	0
**Disease recurrence**	26	8	16	2
**Died**	25	7	16	2
**PFS (months)**				
median	46.25	46.5	44.75	55.25
range	6-132.3	6-125	7-132.3	6–96
**OS (months)**				
median	50	49	50	55.25
range	4.7-132.3	13–125	4.7-132.3	14–96

Note: LMS: leiomyosarcoma; ESS: endometrial stromal sarcoma; CA125: carbohydrate antigen 125; CDFI: color Doppler flow imaging; FIGO: International Federation of Gynecology and Obstetrics; LVSI: lymphovascular space invasion; PFS: Progression-free survival; OS: Overall survival

### Univariate analysis of the total cohort

Among the total 75 patients, 26 (34.67%) patients recurred at the end of follow-up, with 25 dying from recurrence. Recurrence characterized as pelvic mass mostly occurred in pelvic cavity.

The mean PFS and OS were 89.41 (95% confidence interval (CI): 76.07-102.75) and 94.03 (95% CI: 81.67-106.38) months, respectively. The corresponding 5-year PFS and OS rates were 66.5% and 71%, respectively. Compared to patients in advanced stage (FIGO II-IV), patients in stage I had a significantly better prognosis, with longer PFS (98.6 vs. 38.2 months, *P* = 0.001) and a higher 5-year PFS rate (74.7% vs. 27.3%, *P* = 0.001). Similarly, stage I patients had significantly longer OS (103.8 vs. 49 months, *P* < 0.001) and a higher 5-year OS rate (80% vs. 34.1%, *P* < 0.001) compared to their advanced-stage counterparts.

Table 2 showed the comparisons of survival outcomes among various clinicopathological parameters and treatments of the total patients using the log-rank tests. The results revealed that diagnosis age > 50 years, post-menopause, advanced stage and ≥ 1/2 myometrial invasion were associated with shorter PFS and OS (all *P* < 0.05). In addition, LVSI was significantly linked with shorter OS (*P* < 0.001) and marginally significantly shorter PFS (*P* = 0.071).

**Table 2:** Univariate analysis for PFS and OS using the log-rank tests in total cohort and subgroups

	Total cohort						ESS						LMS					
	PFS			OS			PFS			OS			PFS			OS		
	5-year rate (%)	mean	*P*	5-year rate (%)	mean	*P*	5-year rate (%)	mean	*P*	5-year rate (%)	mean	*P*	5-year rate (%)	mean	*P*	5-year rate (%)	mean	*P*
**All**	66.5	89.41		71.0	94.03		80.4	99.56		84.9	103.69		52.2	76.05		58.3	83.46	
**Age at diagnosis**																		
≤ 50	80.6	105.7	0.004	82.7	110.3	0.005	90.5	115.0	0.022	88.8	114.6	0.108						
>50	46.9	62.6		55.1	73.8		66.0	77.5		80.0	92.9							
≤ 51													72.2	94.8	0.022	77.8	103.3	0.006
>51													15.0	37.5		24.4	47.7	
**Menopausal state**																		
premenopausal	78.5	102.4	0.001	82.1	106.2	0.001	91.3	111.3	0.022	90.6	114.8	0.057	68.2	93.0	0.001	76.7	100.0	<0.001
postmenopausal	41.1	50.3		45.0	55.3		60.6	48.5		74.0	55.1		0	23.0		0	31.0	
**CDFI**																		
No	81.6	89.2	0.289	85.6	93.9	0.191	71.6	80.5	0.341	77.9	87.7	0.405	90.0	92.1	0.046	90.0	93.1	0.098
yes	57.9	85.1		65.7	90.4		88.2	111.6		94.1	109.8		29.2	54.6		42.2	69.2	
unknown	66.6	76.3		65.8	83.4		75.0	87.4		72.9	96.7		53.3	62.1		53.3	66.6	
**Tumor size (cm)**																		
<5	92.9	94.7	0.093	92.9	95.3	0.2	100.0	/	0.277	100.0	/	0.406	100.0	/	0.31	100.0	/	0.499
≥ 5	54.1	80.7		61.7	86.6		71.4	/		79.5	/		40.3	/		49.1	/	
miss	84.6	92.6		84.6	101.1		83.3	/		83.3	/		85.7	/		85.7	/	
**FIGO Stage**																		
I	74.7	98.6	0.001	80.0	103.8	<0.001	83.6	103.1	0.177	85.4	109.4	0.252	67.8	94.4	<0.001	77.4	101.0	<0.001
II-IV	27.3	38.2		34.1	49.0		60.0	69.0		80.0	75.8		0	12.6		0	28.3	
miss	0	24.0		0	31.0								0	24.0		0	31.0	
**Pathological type**																		
ESS	80.4	99.6	0.131	84.9	103.7	0.202	/	/		/	/		/	/		/	/	
LMS	52.2	76.1		58.3	83.5		/	/		/	/		/	/		/	/	
Adenosarcoma	66.7	66		66.7	69.2		/	/		/	/		/	/		/	/	
**Histologic grade**																		
low	79.6	99.1	0.137	86.2	104.7	0.097	90.2	110.5	0.003	94.1	118.3	0.001	60.6	74.4	0.059	71.6	80.1	0.026
high	52.2	59.8		53.1	62.7		75.0	52.0		82.5	56.4		0	27.7		0	37.5	
miss	58.8	83.9		64.7	92.3		33.3	21.2		33.3	26.2		66.7	95.2		75.0	105.2	
**Myometrial invasion**																		
<1/2	92.2	116.3	0.001	96.4	122.3	0.001	100.0	120.3	0.016	100.0	124.0	0.064	88.9	114.8	0.069	100.0	127.0	0.012
≥ 1/2	44.8	55.1		52.8	62.2		57.1	59.0		70.1	67.1		35.3	48.1		41.2	55.8	
unknown	74.1	82.7		69.4	80.9		85.7	92.7		85.7	93.1		53.3	32.3		40.0	34.4	
**LVSI**																		
no	67.3	90.6	0.071	72.2	95.4	<0.001	81.0	/	0.005	85.9	/	<0.001	54.1	79.0	0.178	60.8	87.0	0.103
yes	66.7	73.5		66.7	75.2		100.0	/		/	/		33.3	28.2		33.3	31.5	
miss	0	11		0	14		0	/		0	/							
**Treatment plan**																		
surgery	50.7	/	0.615	48.2	/	0.305	81.8	59.7	0.999	81.8	60.5	0.554	25	39.8	0.32	25.0	47.2	0.212
surgery + adjuvant therapy	68.9	/		75.7	/		80.0	99.6		86.9	105.7		58.4	81.4		66.1	88.9	
**Lymphadenectomy**																		
no	62.6	85.6	0.478	69.7	91.4	0.636	79.8	91.9	0.899	87.2	93.3	0.992	43.9	66.4	0.203	53.6	78.3	0.435
yes	75.0	91.8		74.1	97.3		81.8	99.1		80.8	105.2		70.0	81.5		68.6	81.7	
**Chemotherapy regimen**																		
IAP	51.4	56.9	0.241	64.0	64.2	0.224	71.4	/	0.74	80.0	/	0.909	42.9	45.7	0.464	57.1	53.5	0.218
PT	77.0	100.0		82.1	105.9		81.3	/		87.5	/		68.2	91.6		72.7	100.5	
other	75.0	31.1		75.0	32.6		100.0	/		/	/		50.0	16.8		50.0	19.8	

**Note: & age categorization was based on the mean age of each group.** PFS: Progression-free survival; OS: Overall survival; CA125: carbohydrate antigen 125; CDFI: color Doppler flow imaging; FIGO: International Federation of Gynecology and Obstetrics; LMS: leiomyosarcoma; ESS: endometrial stromal sarcoma; LVSI: lymphovascular space invasion; IAP: Ifosfamide + epirubicin + Cisplatin; PT: Paclitaxel + platinum

### Univariate analysis of subgroups

The subgroup analysis of our study differentiated between 36 cases of ESS, 33 LMS and 6 adenosarcomata. Univariate analysis was only performed for the ESS and LMS groups due to the small sample sizes of the adenosarcoma group, and the results were shown in Table 2.

In the ESS group, 31 patients were classified with stage I and 5 with stage II-IV. 8 (22.22%) patients underwent disease recurrence and 7 (19.44%) deaths reported at the last follow-up. The mean PFS and OS were 99.56 (95% CI: 84.48-114.63) and 103.69 (95% CI: 88.73-118.64) months, respectively. The corresponding 5-year PFS and OS rates were 80.4% and 84.9%, respectively. Univariate analysis showed that diagnosis age ≤ 50 years, pre-menopause, low-grade, < 1/2 myometrial invasion, and absence of LVSI were associated with significantly longer PFS, and high-grade and LVSI were related to significantly shorter OS (all *P* < 0.05). Age at diagnosis, menopausal state and myometrial invasion were not significantly associated with OS (*P* > 0.05).

ESS included low-grade ESS (LG-ESS), high-grade ESS (HG-ESS) and undifferentiated uterine sarcoma (UUS) [1]. In our study, there were 21 cases of LG-ESS, 12 cases of HG-ESS, 3 cases of unknown grade and 0 case of undifferentiated sarcoma. PFS was significantly higher in LG-ESS cohort (110.5months) than that in HG-ESS (52 months) and unknown grade (21.2 months) (*P* = 0.003). The OS was also significantly better in LG-ESS patients (118.03 months) than that for HG-ESS (56.4 months) and unknown group (26.2 months) (*P* = 0.001). Meanwhile, patients with unknown grade uterine sarcoma showed the worst prognosis than the other two subtypes.

The LMS group included 24 patients with stage I, 6 with stage II-IV, and 3 unclassified. Recurrence and mortality were reported in 16 patients (48.48%,16/33). The mean PFS and OS were 76.05 (95% CI: 55.87–96.23) and 83.46 (95% CI: 64.94-101.98) months, respectively. The 5-year PFS and OS rates were 52.2% and 58.3%, respectively. Univariate analysis identified that diagnosis age > 51 years, post-menopause and advanced stage as predictors of shorter PFS and OS (*P* < 0.05). Positive CDFI signal was significantly associated with decreased PFS (*P* < 0.05) and marginally with reduced OS (*P* = 0.098). Low-grade and < 1/2 myometrial invasion were significantly associated with longer OS (*P* < 0.05), while only marginally significantly associated with longer PFS.

Comparatively, the ESS subgroup exhibited more favorable outcomes than those of the LMS group, including a significantly lower recurrence rate (22.22% vs. 48.48%, *P* = 0.022), significantly longer PFS (99.56 vs. 76.05 months, *P* = 0.043), and a trend towards longer OS (103.69 vs. 83.46 months, *P* = 0.077), underscoring the variable prognosis and treatment response across uterine sarcoma.

### Multivariable analysis

**Fig. 2:**
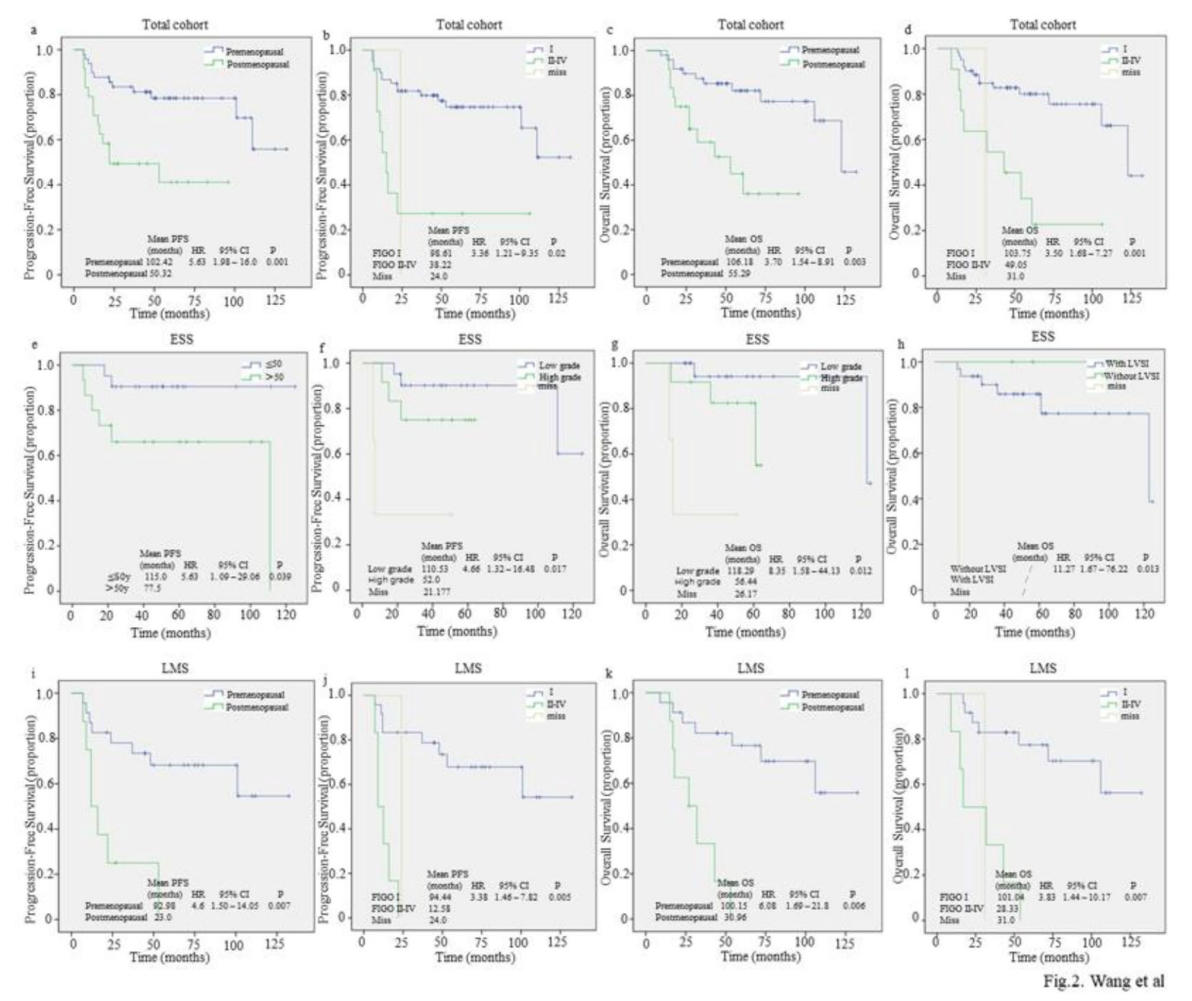
Kaplan-Meier survival curves for progression-free survival (PFS) and overall survival (OS) stratified by clinical and pathological parameters in a-d: total cohort, e-h: endometrial stromal sarcoma (ESS) and i-k: leiomyosarcoma (LMS). **a-b** were stratified by menopausal status and FIGO stage for PFS in the total cohort; **c-d** were stratified by menopausal status and FIGO stage for OS in the total cohort; **e-f** were stratified by age at diagnosis and histological grade for PFS in the ESS group; **g-h** was stratified by histological grade and lymphovascular space invasion (LVSI) for OS in ESS group; **i-j** were stratified by menopausal status and FIGO stage for PFS in LMS group; **k-l** were stratified by menopausal status and FIGO stage for OS in LMS group

The results of the multivariable analysis were revealed in Table 3; Fig. 2, highlighting the independent prognostic risk factors affecting survival within our patient cohort. For the entire cohort, post-menopause (HR = 3.861, 95% CI: 1.65–9.02, *P* = 0.002 for PFS and HR = 3.703, 95% CI: 1.54–8.91, *P* = 0.003 for OS) and advanced stage (HR = 3.418, 95% CI: 1.72–6.79, *P* < 0.001 for PFS and HR = 3.498, 95% CI: 1.68–7.27, *P* = 0.001 for OS) were independent prognostic risk factors for survival.

Similarly, in the LMS subgroup analysis, post-menopause (HR = 4.595, 95% CI:1.50-14.05, *P* = 0.007 for PFS and HR = 6.078, 95% CI:1.69–21.80, *P* = 0.006 for OS) and advanced stage (HR = 3.376, 95% CI:1.46–7.82, *P =* 0.005 for PFS and HR = 3.829, 95% CI:1.44–10.17, *P* = 0.007 for OS) were independent prognostic risk factors of survival, underscoring similar patterns of risk across different sarcoma types.

For the ESS group, diagnosis age > 50 years (HR = 5.627, 95%CI:1.09–29.06, *P* = 0.039) and high-grade (HR = 4.660, 95%CI:1.32–16.48, *P* = 0.017) were found to be independent risk factors of influencing PFS. Furthermore, high-grade (HR = 8.349, 95%CI:1.58–44.13, *P* = 0.012) and the presence of LVSI (HR = 11.266, 95%CI:1.67–76.22, *P* = 0.013) were identified as independent risk factors of OS.

These findings suggested that certain clinicopathological features, particularly menopausal status, disease stage, histological grade and LVSI, played a critical role in predicting the prognosis of patients with uterine sarcoma.

**Table 3:** Multivariate analysis for PFS and OS using Cox-proportional hazards regression models in total cohort and subgroups

Cohort			HR	95% CI	*P*
**total**	**PFS**	**Menopause state**	3.861	1.65–9.02	0.002
		**FIGO stage**	3.418	1.72–6.79	<0.001
	**OS**	**Menopause state**	3.703	1.54–8.91	0.003
		**FIGO stage**	3.498	1.68–7.27	0.001
**subgroups**					
**ESS**	**PFS**	**Age at diagnosis**	5.627	1.09–29.06	0.039
		**Histologic grade**	4.660	1.32–16.48	0.017
	**OS**	**Menopause state**	8.252	0.78–87.46	0.080
		**Histologic grade**	8.349	1.58–44.13	0.012
		**Lymphovascular space invasion**	11.266	1.67–76.22	0.013
**LMS**	**PFS**	**Menopause state**	4.595	1.50–14.05	0.007
		**FIGO stage**	3.376	1.46–7.82	0.005
	**OS**	**Menopause state**	6.078	1.69–21.80	0.006
		**FIGO stage**	3.829	1.44–10.17	0.007

## Discussion

Previous western studies, conducted almost entirely including carcinosarcoma, explored the relationships between risk factors, adjuvant treatments, and survival based on traditional classification systems [12–16]. However, there have been a few studies with respect to them in China and the patient grouping studied by newer classifications excluding carcinosarcoma. In addition, the incidence of uterine sarcoma varies by race and is higher in African-Americans than in other races [3, 17]. Our research presented is a comprehensive analysis of uterine sarcoma and examines 75 Chinese patients over a 10-year period using the latest classification system for more detailed results.

Our findings revealed that LMS had a poorer prognosis than that of ESS. Specifically, for the ESS group, we determined post-menopausal, high-grade and LVSI as key factors associated with reduced survival. Furthermore, the study highlighted the potential diagnostic value of CDFI, enabling it to distinguish between benign and malignant tumors and representing a new research topic.

CDFI provide a new direction for distinguishing uterine sarcoma from uterine fibroids. In our research, 62.9% of patients had CDFI during the examination. CDFI positive signals had a negative effect on survival of LMS group. This suggests the potentially important role of CDFI in the evaluation of the malignant transformation of uterus myoma, which is similar to a finding reported by Yang Hua [18]. Asim Kurjak reported that a cutoff in resistance index (RI) of 0.4 of tumoral blood vessels could distinguish uterine sarcoma from uterus myoma with a higher RI [19]. The diagnostic value of this cutoff is 90.91%, 99.82%, 71.43% and 99.96% in sensitivity, specificity, positive predictive value and negative predictive value, respectively [19]. However, the study had small sample sizes (*n* = 10). Sun et al. found that increased vascularity on color Doppler ultrasound could sometimes favor malignancy, especially when combined with a large size and degenerative cystic changes [20]. Further studies are needed to figure out the clinical significance of CDFI for uterine sarcoma.

Univariate analysis demonstrated that over 50 years, post-menopause, advanced stage and ≥ 1/2 myometrial invasion were significantly associated with poorer survival; while multivariable analysis identified that post-menopause and advanced stage were independent prognostic factors for survival of the total cohort and the LMS group. These findings were consistent with previous studies [14, 21–23]. Thus, they were not limited by race or traditional classification systems.

Studies showed that prognoses of different pathological types of uterine sarcoma varied a lot [24–27]. Our study showed that ESS had a significantly lower recurrence and a higher 5-year survival rate than LMS, and the survival of LG-ESS is prior to those of LMS and HG-ESS, which was in agreement with previous studies [28–31]. It’s reported that pathological subtype is a significant prognostic factor for OS [30]. However, pathological type isn’t a prognostic factor for survival in our study, which may be related to the merger of LG-ESS and HG-ESS into ESS because of small sample size. The proportion of FIGO I stage in the ESS group is higher than that in the LMS group (86.11% vs. 72.73%). Otherwise, misdiagnosis of LMS as uterine leiomyoma because of the same symptoms and delayed treatment due to minimally invasive therapy as well as inadvertent dissemination [32]. Alexandra Huss’s study reported that three cases LMS were diagnosed at tumor recurrence [30]. LG-ESS grew slowly and had a good prognosis in initial stages than that of HG-ESS in our study. Recently several studies have shown that chromosomal rearrangments and gene amplifications can provide new ideas for the diagnosis and treatment of HG-ESS [33, 34], However, our study lacks exploration on relevant molecular markers because of the earlier diagnosed patients.

Compared with previous studies, our investigation is distinctive in several aspects. (1) Geographical and racial backgrounds. Our study is based on certain Chinese patients in a way. It thus considerably complements the currently available literatures that focus on the clinicopathological features and prognosis of uterine sarcomas conducted in Western population. It significantly increases the global knowledge database regarding potential ethnic and geographic differences in sarcomas. (2) Utilization of the new classification system. This study uses the new classification of uterine sarcomas from 2014 in an attempt to provide an up-to-date exploration of their clinicopathological characteristics and prognosis. (3) Detailed subtype analysis. Given the evident heterogeneity within uterine sarcomas, subgroup analysis was performed separately and most survival factors were developed for each sarcoma group, namely ESS and LMS respectively. This is a valuable supplement that should be deserving of clinical attention because different pathological types of sarcomas present their exclusive influential factors and prognoses, which agents nation planning and judgement prognosis. (4) The usage of CDFI, as a potential malignant myoma indicator, offer an innovation that it may play an important role in novel non-invasive diagnostic techniques.

Our study had some limitations. First, the small sample sizes of adenosarcoma limits the statistical power and generalizability of the findings. This constraint makes it challenging to conduct comprehensive subgroup analyses or to conclude the prognostic implications for rare sarcoma types definitively. Second, the retrospective nature of the study design made it subject to selection and recall bias. These biases could affect accuracy of the collected data and interpretation of the study’s findings. Third, the study was performed in a single center. Its findings may not be broadly applicable to all populations. Forth, the study suggests CDFI as a potentially valuable tool for identifying malignant myomas, which is preliminary and requires further validation through larger, prospective studies to determine its clinical utility and accuracy.

## Conclusion

According to our results, LMS is more aggressive than ESS. Post-menopause and advanced stage are independent risk factors of survival for the total patients and LMS, which were not limited to race or traditional classification system. Meanwhile, post-menopause, high-grade and LVSI are independently related to decreased survival in the ESS group. Uterine myoma with blood flow signal may be a useful indicator of malignant myoma, which needs to further validate its diagnostic utility in large-scale, multi-center studies and refine protocols for the management of uterine sarcomas.

## Data Availability

No datasets were generated or analysed during the current study.

## Abbreviations

PFS: Progression-free survival
OS: Overall survival
CS: Carcinosarcoma
ESS: Endometrial stromal sarcomas
LMS: Leiomyosarcomas
UUS: Undifferentiated sarcoma
LG-ESS: Low-grade ESS
HG-ESS: High-grade ESS
FIGO: International Federation of Gynecology and Obstetrics
CA125: Cancer antigen 125
TH-BSO: Total hysterectomy with bilateral salpingo-oophorectomy
CDFI: Color Doppler flow imaging
LVSI: Lymphovascular space invasion
SD: Standard deviations
PT: Paclitaxel and platinum
IAP: Ifosfamide + epirubicin + cisplatin
CI: Confidence interval

## Declarations

### Statement

All methods were carried out in accordance with relevant guidelines and regulations in the study.

## References

[1] Desar IME, Ottevanger PB, Benson C, van der Graaf WTA (2018). Systemic treatment in adult uterine sarcomas. Crit Rev Oncol Hematol.

[2] Mbatani N, Olawaiye AB, Prat J (2018). Uterine sarcomas. Int J Gynaecol Obstet.

[3] Hosh M, Antar S, Nazzal A, Warda M, Gibreel A, Refky B (2016). Uterine sarcoma: analysis of 13,089 cases based on Surveillance, Epidemiology, and end results database. Int J Gynecol Cancer.

[4] Santos P, Cunha TM (2015). Uterine sarcomas: clinical presentation and MRI features. Diagn Interv Radiol.

[5] Lentz SE, Zaritsky E, Tucker LY, Lee C, Lazo IM, Niihara A, Yamamoto M, Raine-Bennett T (2020). Prediction of Occult Uterine Sarcoma before Hysterectomy for women with leiomyoma or abnormal bleeding. J Minim Invasive Gynecol.

[6] Wais M, Tepperman E, Bernardini MQ, Gien LT, Jimenez W, Murji A (2017). A Multicentre Retrospective Review of clinical characteristics of Uterine Sarcoma. J Obstet Gynaecol Can.

[7] Kho KA, Lin K, Hechanova M, Richardson DL (2016). Risk of Occult Uterine Sarcoma in Women undergoing hysterectomy for Benign indications. Obstet Gynecol.

[8] Bi Q, Xiao Z, Lv F, Liu Y, Zou C, Shen Y (2018). Utility of clinical parameters and multiparametric MRI as predictive factors for differentiating uterine sarcoma from Atypical Leiomyoma. Acad Radiol.

[9] Moinfar F, Azodi M, Tavassoli FA (2007). Uterine sarcomas. Pathology.

[10] Rojas C, Tian C, Powell MA, Chan JK, Bateman NW, Conrads TP, Rocconi RP, Jones NL, Shriver CD, Hamilton CA (2020). Racial disparities in uterine and ovarian carcinosarcoma: a population-based analysis of treatment and survival. Gynecol Oncol.

[11] Li N, Wu LY, Zhang HT, An JS, Li XG, Ma SK (2008). Treatment options in stage I endometrial stromal sarcoma: a retrospective analysis of 53 cases. Gynecol Oncol.

[12] Huss A, Klar M, Hasanov MF, Juhasz-Boss I, Bossart M. Prognostic factors and survival of patients with uterine sarcoma: a German unicenter analysis. Arch Gynecol Obstet 2022. 10.1007/s00404-022-06515-2 PMC9984332 35780401

[13] Sucu M, Kucukgoz Gulec U, Paydas S, Guzel AB, Kilic Bagir E, Vardar MA (2021). Clinicopathologic characteristics and prognosis comparison of the uterine high grade endometrial carcinomas. Ginekol Pol.

[14] Cabrera S, Bebia V, Acosta U, Franco-Camps S, Manalich L, Garcia-Jimenez A, Gil-Moreno A (2021). Survival outcomes and prognostic factors of endometrial stromal sarcoma and undifferentiated uterine sarcoma. Clin Transl Oncol.

[15] Ayhan A, Gungorduk K, Khatib G, Firat Cuylan Z, Boran N, Gokcu M, Celik H, Ozgul N, Akbayir O, Simsek T (2021). Prognostic factors and survival outcomes of women with uterine leiomyosarcoma: a Turkish uterine Sarcoma Group Study-003. Curr Probl Cancer.

[16] Chantharasamee J, Wong K, Potivongsajarn P, Qorbani A, Motamed N, Brackert S, Cohen J, Chmielowski B, Kalbasi A, Rao J (2022). Retrospective analysis of adjuvant treatment for localized, operable uterine leiomyosarcoma. Cancer Med.

[17] Kapp DS, Shin JY, Chan JK (2008). Prognostic factors and survival in 1396 patients with uterine leiomyosarcomas: emphasis on impact of lymphadenectomy and oophorectomy. Cancer.

[18] Yang H, Li XC, Yao C, Lang JH, Jin HM, Xi MR, Wang G, Wang LW, Hao M, Ding Y (2017). Proportion of uterine malignant tumors in patients with laparoscopic myomectomy: a National Multicenter Study in China. Chin Med J (Engl).

[19] Kurjak A, Kupesic S, Shalan H, Jukic S, Kosuta D, Ilijas M (1995). Uterine sarcoma: a report of 10 cases studied by transvaginal color and pulsed Doppler Sonography. Gynecol Oncol.

[20] Sun S, Bonaffini PA, Nougaret S, Fournier L, Dohan A, Chong J, Smith J, Addley H, Reinhold C (2019). How to differentiate uterine leiomyosarcoma from leiomyoma with imaging. Diagn Interv Imaging.

[21] Durnali A, Tokluoglu S, Ozdemir N, Inanc M, Alkis N, Zengin N, Sonmez OU, Kucukoner M (2012). Anatolian Society of Medical O: prognostic factors and treatment outcomes in 93 patients with uterine sarcoma from 4 centers in Turkey. Asian Pac J Cancer Prev.

[22] Ishidera Y, Yoshida H, Oi Y, Katayama K, Miyagi E, Hayashi H, Shigeta H (2019). Analysis of uterine corporeal mesenchymal tumors occurring after menopause. BMC Womens Health.

[23] Momtahan M, Emami F, Sari Aslani F, Akbarzadeh-Jahromi M (2020). Evaluation of treatment results and prognostic factors of uterine sarcoma: a single-center experience. J Chin Med Assoc.

[24] Amant F, Mirza MR, Koskas M, Creutzberg CL (2018). Cancer of the corpus uteri. Int J Gynaecol Obstet.

[25] Thangappah RBP (2019). Uterine sarcoma: a clinico-pathological study. J Obstet Gynaecol India.

[26] Li K, Yin R, Li L, Wang D, Li L, Ma C, Ren Q, Wang G, Fan Y, Zhou H (2021). Diagnosis and treatment of uterine sarcoma: a multicenter, real-world study in western China. Med (Baltim).

[27] Burghaus S, Halmen S, Gass P, Mehlhorn G, Schrauder MG, Lux MP, Renner SP, Beckmann MW, Hein A, Thiel FC (2016). Outcome and prognosis in uterine sarcoma and malignant mixed mullerian tumor. Arch Gynecol Obstet.

[28] Park JY, Kim DY, Suh DS, Kim JH, Kim YM, Kim YT, Nam JH (2008). Prognostic factors and treatment outcomes of patients with uterine sarcoma: analysis of 127 patients at a single institution, 1989–2007. J Cancer Res Clin Oncol.

[29] Wang L, Li S, Zhang Z, Jia J, Shan B (2020). Prevalence and occult rates of uterine leiomyosarcoma. Med (Baltim).

[30] Huss A, Klar M, Hasanov MF, Juhasz-Boss I, Bossart M (2023). Prognostic factors and survival of patients with uterine sarcoma: a German unicenter analysis. Arch Gynecol Obstet.

[31] Nordal RN, Kjorstad KE, Stenwig AE, Trope CG (1993). Leiomyosarcoma (LMS) and endometrial stromal sarcoma (ESS) of the uterus. A survey of patients treated in the Norwegian Radium Hospital 1976–1985. Int J Gynecol Cancer.

[32] Sizzi O, Manganaro L, Rossetti A, Saldari M, Florio G, Loddo A, Zurawin R, van Herendael B, Djokovic D (2018). Assessing the risk of laparoscopic morcellation of occult uterine sarcomas during hysterectomy and myomectomy: literature review and the ISGE recommendations. Eur J Obstet Gynecol Reprod Biol.

[33] Micci F, Heim S, Panagopoulos I (2021). Molecular pathogenesis and prognostication of low-grade’’ and high-grade endometrial stromal sarcoma. Genes Chromosomes Cancer.

[34] Kommoss FK, Chang KT, Stichel D, Banito A, Jones DT, Heilig CE, Frohling S, Sahm F, Stenzinger A, Hartmann W (2020). Endometrial stromal sarcomas with BCOR-rearrangement harbor MDM2 amplifications. J Pathol Clin Res.

